# PLGA - encapsulated harmine derivative H-2-168: A promising therapeutic agent for mitigating liver damage in hepatic hydatid disease

**DOI:** 10.1371/journal.pntd.0014483

**Published:** 2026-07-24

**Authors:** Ayinula Tuohetali, Bei Chen, Qinwei Xu, Jiang Zhu, Zhenping Zhang, Xia Chen, Yixueying Ma, Kadierya Kuerban, Kalibixiati Aimulajiang, Huijing Gao

**Affiliations:** 1 State Key Laboratory of Pathogenesis, Prevention and Treatment of High Incidence Diseases in Central Asia, Department of Pharmacy, The First Affiliated Hospital of Xinjiang Medical University, Urumqi, China; 2 College of Veterinary Medicine, Xinjiang Agricultural University, Urumqi, China; 3 Department of Abdominal Surgery, The Third People’s Hospital of Xinjiang Uygur Autonomous Region, Urumqi, China; 4 Clinical Medicine Institute, The First Affiliated Hospital of Xinjiang Medical University, Urumqi, China; University of Buea, CAMEROON

## Abstract

Harmine (HM), a bioactive alkaloid, exhibits remarkable antiparasitic potency. Nevertheless, its translational potential is substantially constrained by neurotoxicity, necessitating the development of safer analogs. H-2-168, a rationally designed derivative synthesized via targeted structural modifications of the HM core, has emerged as a leading candidate for further therapeutic exploration due to its favorable pharmacological profile. To optimize its pharmacokinetic properties and enhance treatment efficacy, this study reports the fabrication of H-2-168-loaded poly (lactic - co - glycolic acid) (PLGA) nanoparticles (H8-PLGA-NPs). Results demonstrated that H8-PLGA-NPs exhibited uniform spherical morphology with an average diameter of 198 nm (PDI = 0.15) and a high drug encapsulation efficiency of 88.6%. Following 30-day oral administration (100 mg/kg/day), H8-PLGA-NPs demonstrated significantly enhanced anthelmintic efficacy and tissue regenerative capacity compared to free H-2-168, while effectively ameliorating inflammatory responses and hepatic fibrosis progression. Notably, systemic toxicity was substantially reduced, particularly hepatotoxicity and cytotoxicity. This study substantiates that PLGA-based nanocarriers markedly improve the therapeutic outcomes of H-2-168 against cystic echinococcosis (CE) through optimized drug encapsulation and delivery efficiency, thereby proposing a promising therapeutic strategy for CE management. Nevertheless, comprehensive investigations into the long-term biosafety profiles of these nanoparticles and optimal dosing regimens warrant further exploration.

## Author summary

Echinococcosis is a severe chronic zoonotic parasitic disease caused by infection with *Echinococcus granulosus (E. granulosus)*, with the liver being the most commonly affected organ. Clinically, hepatic echinococcosis is commonly cystic. The treatment of hepatic echinococcosis in clinical practice often involves a combination of surgery and medication. Albendazole is the first-line drug recommended by the WHO for anti-echinococcosis therapy, but it suffers from poor absorption, low drug concentrations at the lesion sites, and numerous side effects, highlighting the urgent need to explore new drugs. Previous studies by our research group have shown that the harmine derivative H-2-168 exhibits promising anti-echinococcosis activity, but its bioavailability is very low. PLGA is a safe, biodegradable polymer known for its advantages in enhancing drug bioavailability, targeting, and sustained release. Therefore, in this study, we prepared H8-PLGA-NPs and investigated their therapeutic effects on mice with hepatic cystic echinococcosis. The results demonstrated that H8-PLGA-NPs exhibited superior anti-CE effects compared to H-2-168. Furthermore, H8-PLGA-NPs alleviated inflammatory responses and hepatic fibrosis. These findings suggest that H8-PLGA-NPs hold promise as a potential drug for the treatment of hepatic echinococcosis.

## 1. Introduction

CE caused by the larval stage of the tapeworm *E. granulosus* represents a globally significant zoonotic disease that profoundly impacts human health and livestock production [1,2]. CE exhibits a wide geographical distribution, with endemic regions spanning western China, Central Asia, South America, and Mediterranean countries [3,4]. The World Health Organization (WHO) has designated echinococcosis as one of 17 neglected tropical diseases targeted for control or elimination by 2050 [5]. Current treatment modalities for *E. granulosus* tapeworm infections primarily involve surgical resection and pharmacotherapy, each with significant limitations. Surgical intervention, although effective for cyst removal, is often invasive and entails risks of recurrence and postoperative complications. Conversely, medications such as albendazole (ABZ) exhibit variable efficacy and may be associated with adverse effects [6].

**Fig 1:**
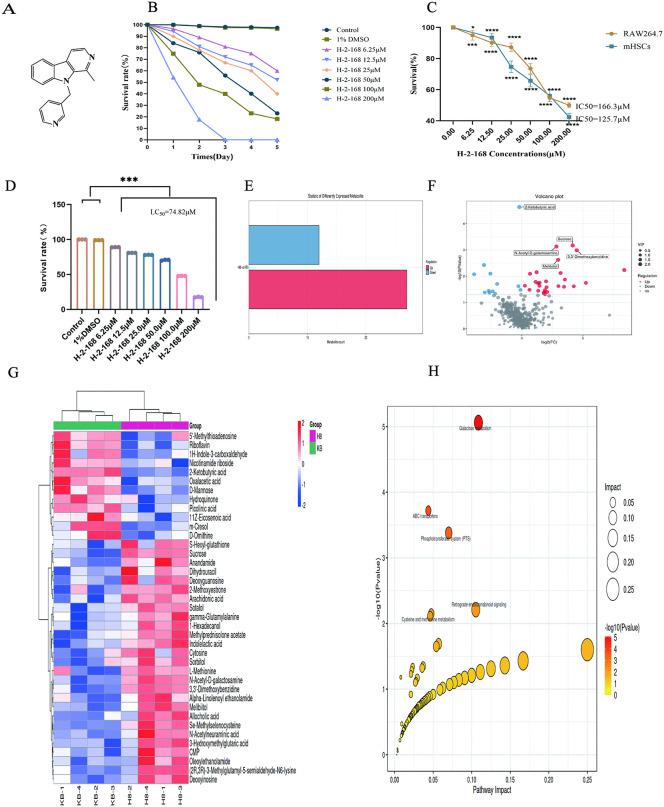
Effect of H-2-168 on PSCs activity and structure. **(A)** Molecular structure of HM derivative H-2-168. **(B)** PSCs’ activity (mean ± SD, n = 3) after 5 days of gradient concentration intervention, 95% CI: 43.34–184.87 μM. **(C)** Effects of H-2-168 on the viability of RAW264.7 and AML-12 cells. **(D)** Statistical graph of metabolite differences, where red indicates metabolite up-regulation and blue indicates metabolite down-regulation. **(E)** Volcano plot of differentially enriched metabolites in the H-2-168 group versus the control group *in vitro*, where red dots indicate metabolite up-regulation, blue dots indicate metabolite down-regulation, and gray dots indicate metabolites that do not meet the criteria for differential screening. **(F)** In the differential metabolite classification heat map, color indicates correlation; red indicates positive correlation, blue indicates negative correlation, and darker indicates stronger correlation. **(G)** Bubble plot showing the KEGG pathway enrichment analysis results of differentially enriched metabolites. The size of the bubbles indicates the number of metabolites associated with the pathway, and the color indicates the p-value. Redder colors indicate lower p-values, while bluer colors indicate higher p-values. Data are shown as mean ± SD (n = 4 independent experiments). Statistical significance is denoted as *P < 0.05, **P < 0.01, ***P < 0.001.

The urgent need for novel and efficacious therapeutics against echinococcosis has driven remarkable advances in anti-parasitic drug discovery. *Peganum harmala L.*, a herb rich in alkaloids, has garnered scientific interest. Harmine (HM), an alkaloid extracted from its seeds and whole plants, demonstrates diverse pharmacological activities, including anti-CE effects [7]. However, the neurotoxicity of HM restricts its clinical translation. We synthesized a derivative, H-2-168 (Fig 1A), to overcome this limitation. H-2-168 effectively decreased the viability of *E. granulosus* and demonstrated higher potency than HM. Oral administration of H-2-168 also exhibited superior efficacy against cysticercosis compared with albendazole and HM [8]. Moreover, H-2-168 showed lower toxicity than HM, characterized by improved absorption profiles and reduced neurotoxicity, indicating its potential as a therapeutic agent for CE.

However, further studies are needed to validate its clinical utility fully. In recent years, PLGA, a biodegradable polymer, has emerged as a research focus in drug delivery systems due to its excellent biocompatibility and tunable degradation rate [9]. It has been demonstrated that amphiphilic block copolymer PEG-PLGA NPs can serve as safe drug nanocarriers, exhibiting sustained-release properties and enhancing in vivo drug bioavailability and stability [10]. Ligand-modified PEG-PLGA NPs can target specific tumor surface receptors, improving tumor-targeting efficiency. Encapsulating sorafenib in PLGA NPs significantly improves drug stability and cellular uptake, potentiates its antitumor effect, and reduces off-target distribution and associated toxicity [11]. In a rat model of carbon tetrachloride-induced hepatic fibrosis, PLGA-encapsulated curcumin exerted stronger hepatoprotective effects and markedly decreased the extent of hepatic fibrosis [12]. In CE treatment, PLGA-loaded nanoparticles enhance therapeutic efficacy by encapsulating antiparasitic drugs for targeted delivery to the infection site [13]. Therefore, the objective of this study was to evaluate the therapeutic efficacy of PLGA-encapsulated H-2-168 nanoparticles in a murine model of *E. granulosus*. We hypothesized that the PLGA nanoparticle formulation would significantly enhance the delivery and bioavailability of H-2-168 at the cyst site, leading to markedly superior parasitocidal effects and cyst damage compared to treatment with free H-2-168.

## 2. Materials and methods

### 2.1 Ethic statement

The study protocol was approved by Xinjiang Medical University’s Experimental Animal Ethics Committee (IACUC 20170420-04).

### 2.2 Animals

Six to eight-week-old female SPF Kunming mice (20 ± 2 g) were obtained from Xinjiang Medical University’s Laboratory Animal Center. All animals were housed in isolation facilities at the university’s Animal Experiment Center.

### 2.3 Preparation of PLGA NPs loaded with H-2-168

H-2-168-loaded PLGA nanoparticles (NPs) were prepared using a double-emulsion (W_1_/O/W_2_) method, as described in a published protocol [14]. Briefly, the inner aqueous phase (W₁) was prepared by dissolving 15 mg of H-2-168 (Xinjiang Huashidan Pharmaceutical Co., Ltd.) in 2 mL of ultrapure water. This solution was then emulsified with 10 mL of chloroform (Xinbote Chemical Co., Ltd.) containing 50 mg of PLGA (Jinan Daigang Biotechnology Co., Ltd., lactide: glycolide = 75:25, Ester-terminated, The intrinsic viscosity is 0.2 dL/g.) using a probe sonicator (Xinzhi Biotechnology, model JY92-IIN, No. 6 extension rod) at 80% amplitude (with a 5 s pulse interval) for 5 min in an ice bath to form the primary W₁/O emulsion. The primary emulsion was subsequently dispersed into 50 mL of an aqueous solution containing 0.2% (w/v) sodium taurocholate (Yuanye Biotechnology Co., Ltd.), followed by a secondary sonication at 100% amplitude for 10 min to form the final double emulsion (W_1_/O/W_2_). The organic solvent (chloroform) was removed by rotary evaporation. The nanoparticles were collected by centrifugation (12,000 × g, 30 min), washed twice with ultrapure water, and lyophilized for long-term storage at -20°C.

### 2.4 Characterization of PLGA NPs loaded with H-2-168

For drug loading analysis, 5 mg H8-PLGA-NPs were dissolved in methanol by ultrasonication, filtered (0.45 μm), and analyzed by HPLC to determine H-2-168 content. Encapsulation efficiency (EE%) was calculated in triplicate as: [(Total drug - Free drug)/Total drug]×100% [15]. Nanoparticle characterization included: (1) Size distribution and zeta potential (NanoZS90 analyzer, Malvern); (2) Morphology by SEM (JSM IT-800) and TEM (JEM-F200). For *in vitro* release studies, 2 mL aliquots of H-2-168 solution and H8-PLGA-NPs (5 mg H-2-168 equivalent) were dialyzed (MWCO 8–14 kDa) against 50 mL PBS at 37 ± 0.5°C (100 rpm). Samples (2 mL) were collected at predetermined intervals (0.5-48 h) with equal PBS replacement, and H-2-168 content was quantified by HPLC to calculate cumulative release (n = 3).

### 2.5 Cells

RAW264.7 cells (2×10^5^ cells/well) were plated in 96-well plates for 24 h of attachment. After PBS washing, cells were treated with H-2-168 or H8-PLGA-NPs (0–200 μM, 4 replicates) for 48). Cell viability was assessed using the CCK-8 kit (Yeasen Biotechnology, Shanghai, 10 μL/well, 2 h incubation), with absorbance measured at 450 nm (Thermo Fisher microplate reader).

### 2.6 Parasite collection and culture

Viable *E. granulosus* sensu lato protoscoleces (PSCs) used in this study were aseptically aspirated from hepatic hydatid cysts of naturally infected sheep. Infected sheep livers were obtained from the Slaughterhouse in Urumqi, Xinjiang Uygur Autonomous Region, China, a well-documented endemic area for cystic echinococcosis. Livers were transported on ice to our laboratory within 4 hours post-slaughter and processed immediately. Protoscoleces (PSCs) were isolated through mechanical grinding, sequential washing, and filtration. After 30 min digestion in 1% pepsin (pH 3.0, 37°C; Servicebio) [16], viability was determined by eosin exclusion (>95% viable PSCs were selected for *in vivo* studies). All procedures involving the handling, centrifugation, and viability assessment of live PSCs were performed within a biological safety cabinet. Personnel wore appropriate personal protective equipment. All liquid and solid wastes containing infectious material were inactivated by autoclaving at 121°C for 30 minutes before disposal. The collection of parasites complied with ethical guidelines under approval number IACUC 20170420-04.

### 2.7 Investigating the *In Vitro* activity of H-2-168 against PSCs

Following 48h acclimatization, viable PSCs (>95%) were plated in 96-well plates (~200 PSCs/well). Treatments included: (1) negative control (0.1% DMSO), and (2) H-2-168 (6.25-100 μM, 2 μL/well in DMSO). Survival rates were assessed daily (days 1–5) by eosin staining (n = 3).

### 2.8 Animal infection model and drug administration in *E. granulosus* -infected mice

Hepatic echinococcosis was established in mice via hepatic portal vein injection of 3000 protoscoleces (PSCs) in 0.2 mL saline. After 4 months of infection, animals were grouped based on body weight and initial cyst burden assessment and subsequently randomized into six groups (n = 5/group) using a computer-generated sequence to ensure balanced baseline characteristics. The groups were: (1) Model control (0.5% CMC-Na), (2) Positive control [Albendazole (ABZ), 50 mg/kg/day in 0.5% CMC-Na], (3) H-2-168 (25 mg/kg/day, aqueous solution), (4) H-2-168 (50 mg/kg/day, aqueous solution), (5) H-2-168 (100 mg/kg/day, aqueous solution), and (6) H8-PLGA-NPs (100 mg/kg/day, aqueous suspension). All treatments were administered orally by gavage once daily for 30 consecutive days under a single-blind design where investigators responsible for dosing and assessment were blinded to group allocation.

### 2.9 Immunohistochemical staining (IHC)

Tissue sections were first deparaffinized and underwent antigen retrieval. To inhibit endogenous peroxidase activity, they were treated with 3% H_2_O_2_ for 15 minutes, followed by blocking with 5% goat serum for 30 minutes. Primary antibodies were incubated overnight at 4°C (16 h). Detection was performed using HRP-conjugated secondary antibodies (1 h, 37°C), followed by DAB chromogen development (3 min) and hematoxylin counterstaining (35 s). Finally, stained sections were imaged using a fluorescence microscope (Olympus, Japan) and analyzed quantitatively with ImageJ.

### 2.10 RNA isolation and quantitative reverse transcription PCR (qRT-PCR)

Liver tissue RNA was isolated using Trizol reagent, followed by cDNA synthesis (PrimeScript RT kit). Gene expression was quantified by qRT-PCR (SYBR Premix Ex Taq II, Applied Biosystems 7500 system). Primer sequences are listed in S1 Table.

### 2.11 Cytokine quantification by luminex multiplex assay

Serum samples from the H-2-168 and H8-PLGA-NPs groups were submitted for analysis to Shanghai Univision Biotechnology Co.

### 2.12 Pharmacokinetics

Twelve SPF SD rats (6 males, 6 females) were equally divided into H-2-168 and H8-PLGA-NPs groups (n = 6/group). Both groups received a single oral dose (50 mg/kg H-2-168 equivalent). Blood samples (0.4 mL) were collected via retro-orbital plexus at predetermined intervals (0.25-24 h) into heparinized tubes. Plasma was separated using centrifuge (Zhongke Zhongjia, China) at 3500 rpm for 15 minutes and processed for analysis: (1) 0.1 mL plasma + 10 μL tinidazole IS (8.16 μg/mL); (2)290 μL acetonitrile addition (vortex 2 min); (3)Centrifugation (12,000 rpm, 15 min, 4°C); HPLC analysis of 200 μL supernatant.

### 2.13 Metabolomics

Serum metabolomics analysis was performed on samples from the PSCs, model, H-2-168, and H8-PLGA-NPs groups. After thawing at 4°C, the samples were homogenized by vortexing. An aliquot of each sample was then combined with 400 μL of methanol, vortexed, and centrifuged at 12,000 rpm for 10 min (4 °C). The supernatant was collected, concentrated, and dried. The dried extract was reconstituted in 80% methanol (containing 4 ppm 2-chloro-L-phenylalanine), filtered through a 0.22-μm membrane, and analyzed by LC-MS. The LC-MS system consisted of a Thermo Vanquish HPLC coupled to a Thermo Orbitrap Exploris 120 mass spectrometer. Metabolite identification was carried out by matching spectral data against HMDB [17], MassBank [18], and KEGG [19] databases. The statistical significance of the P value was obtained by a statistical test between groups. Finally, combined with P value, VIP (OPLS-DA variable projection importance) and FC (multiple of difference between groups) to screen biomarker metabolites. By default, when *P* < 0.05 and VIP > 1, we consider the metabolite to have significant differential expression [20].

### 2.14 Statistical data analysis

Statistical analyses were carried out using GraphPad Prism 8.0 (GraphPad Prism, USA). All data are presented as the mean ± SD. Differences between groups were evaluated by one-way ANOVA.

## 3. Results

### 3.1 H-2-168 significantly inhibits PSCs’ activity *in vitro*

**Fig 2:**
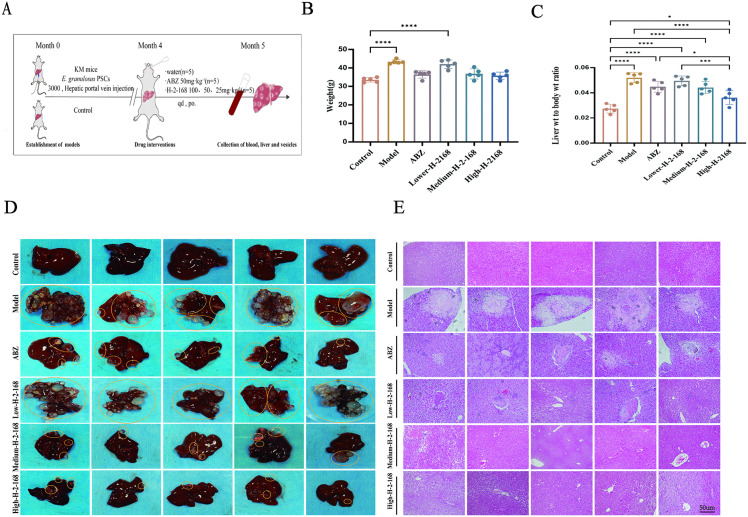
Therapeutic efficacy of H-2-168 in *E. granulosus*-infected mice. **(A)** Experimental timeline showing infection and treatment protocol (n = 5/group). **(B)** Final body weight measurements. **(C)** Liver-to-body mass ratio. **(D)** Representative images of hepatic gross morphology. **(E)** H&E-stained liver sections (scale bar: 50 μm). Data are shown as mean ± SD (n = 5 independent experiments). Statistical significance is denoted as *P < 0.05, **P < 0.01, ***P < 0.001.

H-2-168’s molecular structure is shown in Fig 1A. As shown in Fig 1B, H‑2‑168 significantly reduced PSC viability in a dose‑ and time‑dependent manner. Statistical analysis (one‑way ANOVA with Tukey’s test) confirmed that, starting from day 2, the differences between each treatment group (100 and 200 μM) and the control group were statistically significant (*P* < 0.05). Notably, the inhibitory effect was most pronounced in the 200 μM group, with PSC viability declining to the limit of detection (0%) by day 3, and its effect was significantly stronger than that of the lower‑dose group (*P* < 0.01). Based on nonlinear regression analysis of the dose‑response data, the IC₅₀ of H‑2‑168 was determined to be 74.82 μM(Fig 1D). To evaluate the cytotoxicity of H-2-168 on immune cells (RAW 264.7 macrophages) and hepatic stellate cells (mHSC), cell viability was measured separately using the CCK-8 assay. The results (Fig 1C) showed that H-2-168 significantly inhibited the viability of both cell types in a concentration-dependent manner over 48 hours. The inhibitory effect was most pronounced at 200 μM in both cell lines. Data indicated that mHSC cells were more sensitive to H-2-168 than RAW cells, as evidenced by a greater reduction in cell viability and a lower calculated half-maximal inhibitory concentration (IC₅₀). These findings suggest that H-2-168 exhibits differential cytotoxic effects across different cell lines. LC-MS/MS-based label-free metabolomics identified 443 metabolites in H-2-168-treated and control groups *in vitro*. Among these, 39 metabolites showed differential expression (27 upregulated and 12 downregulated; *P* < 0.05, VIP > 1) (S2 Table). The clustering heatmap (Fig 1G) and KEGG enrichment analysis (Fig 1H) revealed significant alterations in metabolic pathways, including galactose metabolism and ABC transporters, in the H-2-168-treated group compared with the control group.

### 3.2 H-2-168 significantly reduces parasite burden in *E. granulosus*-infected mice

An experimental model was established using KM mice infected with *E.granulosus* (Fig 2A). Body weight changes of different groups were recorded at week 20 (Fig 2B), and liver weight was measured to calculate the liver-to-body weight ratio (Fig 2C). The H-2-168 drug-intervention group exhibited significantly greater therapeutic efficacy compared to the model and ABZ groups. HE staining was performed to evaluate tissue structural changes (Fig 2E). Results revealed that the hepatic tissue architecture of the model group mice was severely damaged, characterized by disorganized hepatocyte arrangement and indistinct hepatic sinusoids. In the ABZ group, although vesicles persisted in liver tissue, they displayed a clear structure with well-defined boundaries, suggesting absorption of vesicular contents. The medium- and high-dose H-2-168 groups (50 and 100 mg/kg) showed more intact liver tissue structures. H-2-168 demonstrated a dose-dependent therapeutic effect within the tested range (25, 50, and 100 mg/kg), with the 100 mg/kg regimen achieving the most pronounced therapeutic outcome.

### 3.3 H-2-168 significantly ameliorates liver function in *E. granulosus*-infected mice

**Fig 3:**
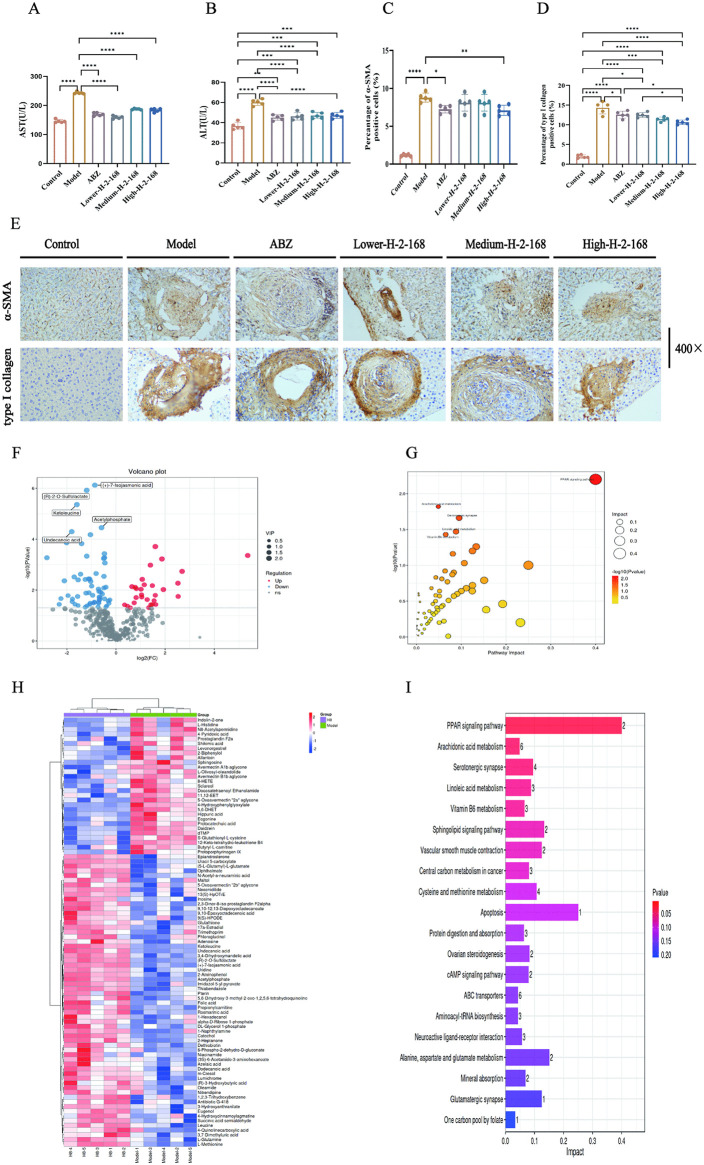
Therapeutic effects of H-2-168 on liver function and metabolic profiles in *E. granulosus*-infected mice. **(A-B)** Serum levels of hepatic injury markers: (A) AST and (B) ALT. **(C-E)** Hepatic fibrosis assessment: Quantitative analysis of (C) α-SMA and (D) collagen I expression by immunohistochemistry; (E) representative IHC images (scale bar: 50 μm). (F-I) Metabolic profiling: **(F)** Volcano plot of differentially expressed metabolites (high-dose H-2-168 vs model group); **(G)** KEGG pathway enrichment analysis (bubble plot); **(H)** hierarchical clustering heatmap of differential metabolites; **(I)** KEGG pathway enrichment bar graph (color gradient represents -log10(p-value), with red indicating greater significance. Data are presented as mean ± SD of n = 5 independent experiments. Statistical significance is denoted as *P < 0.05, **P < 0.01, ***P < 0.001.

Aspartate aminotransferase (AST) and alanine aminotransferase (ALT) serve as sensitive biomarkers of hepatocellular injury. The study revealed that serum AST and ALT levels in the model group were significantly elevated compared to those in the healthy control group (Fig 3A, 3B), providing compelling evidence that CE induces hepatocellular damage. Notably, H-2-168 intervention led to a significant reduction in AST and ALT levels, indicating the drug’s ability to mitigate hepatocellular injury and confer hepatic protection. To further elucidate liver pathological changes, IHC staining was performed on liver lesion sections of mice (Fig 3C). The results demonstrated that the positive area rates of α-SMA and collagen I immunoindicators in the livers of the H-2-168 group were significantly lower than those in the model group (Fig 3D, 3E). Liver metabolites were analyzed to explore the mechanism of echinococcosis. 364 metabolic molecules were screened, of which 91 were differentially expressed (29 upregulated and 62 downregulated) (S3 Table). Volcano plot analysis (Fig 3F) highlighted significant differences in metabolic molecules between the model and H-2-168 groups. Metabolites were filtered based on variable importance in the projection (VIP)>1 and *P* < 0.05. Following quantitative analysis using the Euclidean distance matrix, heat maps of differentially expressed metabolites were generated via the complete linkage method (Fig 3G), demonstrating effective clustering. Metabolite enrichment analysis (Fig 3H, 3I) showed that linoleic acid and arginine metabolic pathways were significantly associated with echinococcosis progression. The bubble size in the bubble plot (Fig 3H) visually represented the impact of each pathway.

### 3.4 Physical properties of H-2-168-loaded NPs

**Fig 4:**
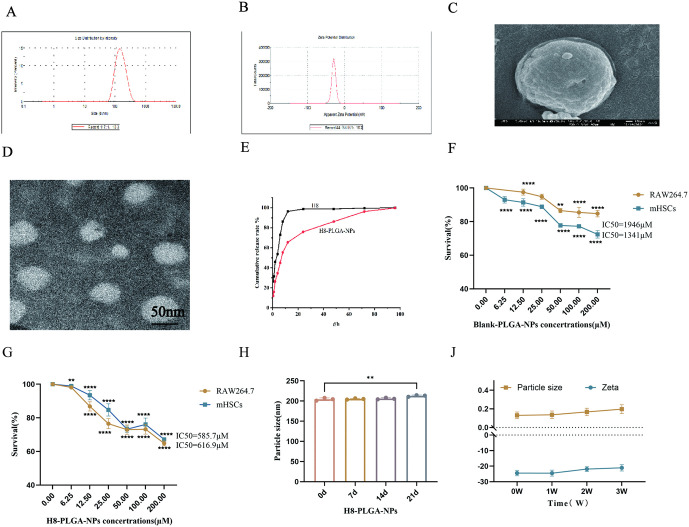
Physicochemical characterization and biocompatibility assessment of H8-PLGA-NPs. **(A)** Hydrodynamic size distribution by dynamic light scattering (DLS). **(B)** Surface charge distribution by zeta potential analysis. **(C)** SEM image demonstrating nanoparticle morphology. **(D)** TEM image showing internal structure (scale bar: 50 nm). **(E)** *In vitro* drug release profile in phosphate-buffered saline (PBS, pH 7.4) at 37°C (n = 3 independent experiments). **(F)** Blank PLGA-NPs and **(G)** H8-PLGA-NPs. Cytocompatibility in RAW264.7 and AML-12 cells was evaluated using the CCK-8 assay after treatment with the respective nanoparticles. Values are mean ± SD (n = 4 independent experiments). **(H)** Particle size stability. The hydrodynamic diameter of H8-PLGA-NPs was monitored over 28 days in vitro (storage at 4°C in PBS). **(J)** Polydispersity index (PDI) stability. The PDI of H8-PLGA-NPs, indicating uniformity in size distribution, was assessed over 28 days in vitro. **(K)** Zeta potential stability. The surface charge (zeta potential) of H8-PLGA-NPs was measured over 28 days in vitro to evaluate colloidal stability. Data are mean ± SD (n = 3 independent experiments). Statistical significance: *P < 0.05, **P < 0.01 and ***P < 0.001.

Following the centrifugal precipitation of nanoparticles (H8-PLGA-NPs), the amount of free H-2-168 in the supernatant was determined. The results indicated that PLGA encapsulated 88.6% of H-2-168. The H8-PLGA-NPs exhibited a mean particle size of 197.3 ± 8.00 nm (Fig 4A), a zeta potential of -28.7 ± 1.17 Mv (Fig 4B), and a PDI of 0.15 ± 0.023. SEM and TEM images (Fig 4C, 4D) revealed that the H8-PLGA-NPs had a spherical morphology with a rough surface. Additionally, the CCK-8 cell viability assay (Fig 4E) demonstrated that the cumulative release of H-2-168 raw material drug reached nearly 100% within 12 h, indicating complete drug release. Conversely, H8-PLGA-NPs exhibited a 65% cumulative release at the 12-hour time point, with continuous slow release observed up to 72 h, confirming their sustained-release characteristics. Through kinetic modeling analysis of the release curve, the results show that the release process of H8-PLGA-NPs conforms to the first-order release kinetic mechanism. In vitro release data. The specific data and analysis are shown in S4 Table. To evaluate the cytotoxicity of H8-PLGA-NPs on immune cells (RAW 264.7 macrophages) and hepatic stellate cells (mHSC), cell viability was measured separately using the CCK-8 assay. The results (Fig 4F) showed that H8-PLGA-NPs significantly inhibited the viability of both cell types in a concentration-dependent manner within 48 hours, with the most pronounced effect observed at 200 μM. Furthermore, RAW cells were more sensitive to H8-PLGA-NPs than mHSC cells, as evidenced by both a greater reduction in cell viability and a lower half-maximal inhibitory concentration (IC₅₀). This suggests that the nanoparticulate formulation exhibits differential cytotoxic effects across different cell lines. In addition, blank PLGA nanoparticles did not induce significant toxicity in either cell type (Fig 4G). The physical stability of H8-PLGA-NPs was evaluated over 21 days in aqueous solution at 4°C by monitoring key physicochemical parameters. The particle size showed excellent stability, with only a marginal increase from 204.40 ± 2.82 nm on day 0 to 212.83 ± 1.62 nm on day 21, and no signs of significant aggregation were observed (Fig 4H). The polydispersity index (PDI) remained consistently low throughout the study, ranging from 0.13 ± 0.02 to 0.20 ± 0.04, which confirmed the maintenance of a homogeneous nanoparticle population (Fig 4J). Similarly, the surface charge, as indicated by the zeta potential, exhibited minimal variation, shifting slightly from -24.43 ± 1.45 mV at the initial point to -21.06 ± 1.65 mV at the endpoint (Fig 4J).

### 3.5 H8-PLGA-NPs attenuate the level of liver fibrosis caused by *E. granulosus* infection

**Fig 5:**
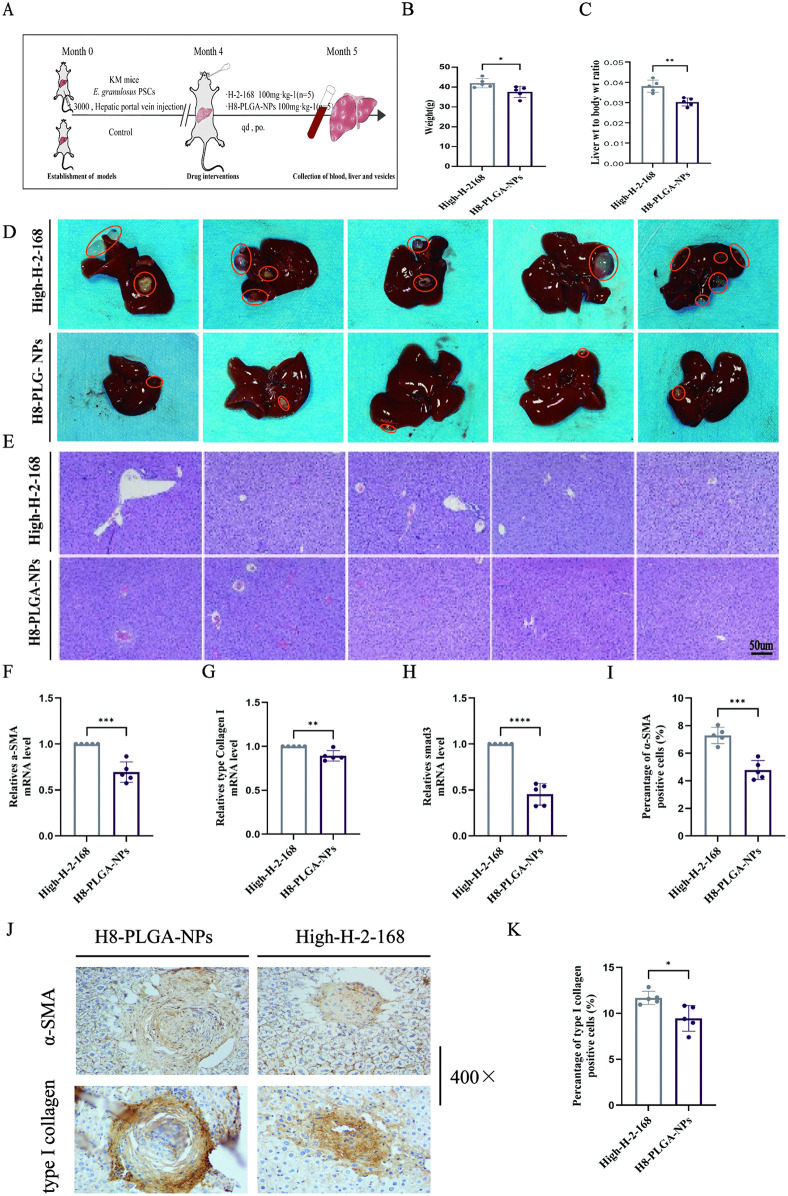
Oral administration of H8-PLGA-NPs reduces *E. granulosus* parasite burden and ameliorates hepatic pathology in infected mice. **(A)** Experimental design: Mice were infected with *E. granulosus* and treated orally with H8-PLGA-NPs (n = 5/group). **(B)** Body weight changes at endpoint. **(C)** Liver-to-body weight ratio. **(D)** Macroscopic liver morphology. **(E)** HE staining of liver sections showing pathological changes. **(F-H)** qRT-PCR analysis of fibrotic markers (α-SMA, collagen I, Smad3) mRNA expression (n = 5). **(I-K)** Quantitative immunohistochemistry analysis of α-SMA and collagen I protein expression; (J) representative IHC images. Data are shown as mean ± SD (n = 5 independent experiments). Statistical significance is denoted as *P < 0.05, **P < 0.01, ***P < 0.001.

An experimental model of KM mice infected with *E. granulosus* was successfully established (Fig 5A). Following one month of oral administration, body weight changes across different groups were recorded (Fig 5B), and liver weights were measured to calculate the liver-to-body weight ratio (Fig 5C). The data demonstrated that the H8-PLGA-NPs group exhibited greater improvement in body weight compared to the H-2-168 group, and the liver - to - body weight ratio in the H8-PLGA-NPs group was closer to the normal level, indicating a more favorable therapeutic effect. HE staining was performed to assess changes in liver tissue structure (Fig 5E). The results revealed that the liver tissue architecture was more intact in the H8-PLGA-NPs group. To further explore the molecular-level regulatory effects of PLGA NPs on liver fibrosis, mRNA levels of fibrosis markers were quantified. Compared with the H-2-168 group, the H8-PLGA-NPs group showed significant reductions in the mRNA expression of α-SMA (Fig 5F), collagen type I (Fig 5G), and Smad3 (Fig 5H) (*P* < 0.05). Furthermore, IHC staining analysis of liver tissue sections (Fig 5I) showed that the protein expression levels of α-SMA and type I collagen were significantly lower in the H8-PLGA-NPs group than in the H-2-168 group (*P* < 0.05, Fig 5I – 5K). These findings further validate the superior efficacy of H8-PLGA-NPs in alleviating liver fibrosis.

### 3.6 H8-PLGA-NPs attenuate hepatic inflammatory counteraction due to *E. granulosus* infection

**Fig 6:**
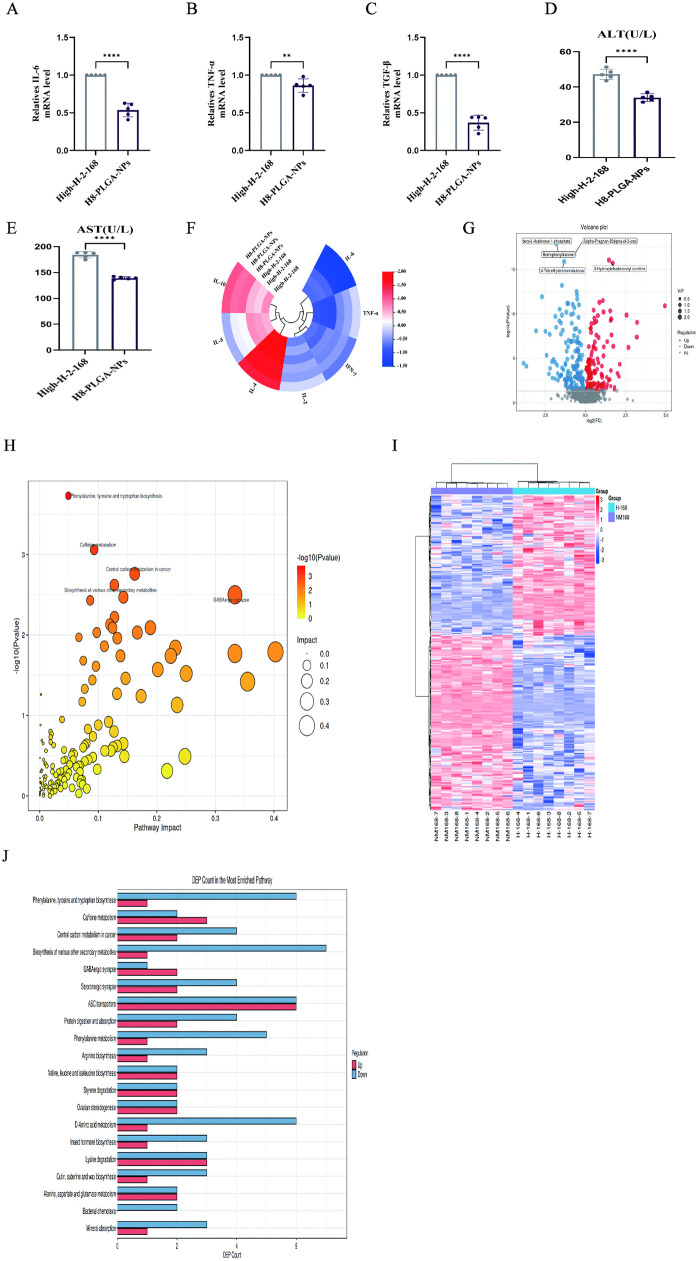
H8-PLGA-NPs attenuate *E. granulosus*-induced inflammatory responses and hepatic injury. **(A-C)** qRT-PCR analysis of IL-6, TNF-α, and TGF-β mRNA levels in liver tissues (n = 5). **(D-E)** Serum AST and ALT levels (n = 5). **(F)** Volcano plot of differentially expressed metabolites. **(G)** KEGG pathway enrichment analysis (bubble plot). **(H)** Heatmap of metabolite profiles. **(I)** Bar graph of enriched KEGG pathways. Data are presented as mean ± SD of n = 5 (panels A–F) or n = 8 (panels G–J) independent experiments. Statistical significance is denoted as *P < 0.05, **P < 0.01, ***P < 0.001.

The pharmacokinetic study results (Table 1) showed that for the H-2-168, the time to peak concentration (*t*_max_) was 0.5 hours, the peak concentration (*C*_max_) was (197 ± 47.66) ng/mL, the half-life (*t*_1/2_) was 1.08 hours, and the plasma concentration fell below the limit of detection (LOD) by 8 hours. In contrast, the H8-PLGA-NPs group exhibited a prolonged *t*_max_ of 1.0 hours, an increased *C*_max_ of (232.70 ± 37.13) ng/mL, a significantly extended *t*_1/2_ of 3.42 hours, with detectable drug levels still present at 24 hours, and the mean residence time (MRT) was substantially prolonged. Calculation of the area under the plasma concentration-time curve (AUC) revealed that the nanoparticle formulation enhanced bioavailability by 2.9-fold compared to the raw drug. These results, together with the chromatographic data presented in S2 Fig, collectively confirm that PLGA nanoparticles significantly improved the sustained-release properties of H-2-168, effectively delaying its elimination and enhancing its absorption efficiency. To explore the modulation of inflammatory responses by PLGA nanoparticles, cytokines were quantified in mouse liver samples. Results (Fig 6A–6C) showed that levels of pro-inflammatory factors, including IL-6, TNF-α, and TGF-β, were significantly lower in the liver tissues of the H8-PLGA-NPs group compared to the H-2-168 group, indicating that the nanoparticles effectively suppressed the inflammatory cascade and reduced liver tissue damage. Safety assessment demonstrated that serum ALT and AST levels were significantly decreased in the H8-PLGA-NPs group relative to the H-2-168 group (Fig 6D, 6E), confirming the favorable *in vivo* biocompatibility of PLGA nanoparticles without apparent hepatotoxicity. Luminex analysis of sample factor expression (Fig 6F) revealed that the H8-PLGA-NPs group exhibited significantly increased IL-4 levels and decreased IFN-γ and IL-1β levels compared to the H-2-168 group, suggesting that the NPs regulated the Th1/Th2 immune balance to promote anti-inflammatory responses. Metabolomics screening identified 893 differential metabolites, with 304 showing significant differential expression (135 upregulated and 169 downregulated) (S5 Table). Five metabolites, including β-α-arabinose phosphate, exhibited significant abundance differences between groups (Fig 6G, 6H. Metabolic pathway enrichment analysis (Fig 6I, 6J) indicated that metabolic changes in the H8-PLGA-NPs group predominantly involved amino acid biosynthesis pathways (e.g., phenylalanine) and caffeine metabolism, potentially related to the PLGA delivery mechanism and providing insights into echinococcosis metabolic characteristics.

**Table 1:** Pharmacokinetic parameter results (n = 6).

Parameters	H-2-168	H-2-168 + PLGA NPs
*C*_max_ (ng/mL)	197 ± 47.66	232.70 ± 37.13
*t*_max_	0.5 ± 0.00	1.00 ± 0.00
*t*_1/2_ (h)	1.08 ± 0.32	3.42 ± 1.36
AUC(ng/mL*h)	338.75 ± 58.20	992.79 ± 262.16
MRT(h)	1.96 ± 0.21	4.35 ± 0.88

## 4. Discussion

This study investigated the therapeutic potential and underlying mechanisms of H-2-168 and its PLGA nanoparticles in treating. Recent research efforts have focused on screening anthelmintic plant compounds from traditional herbal sources, including *Salvadora persica*, *Trametes robiniophila murr*, *Satureja khuzestanica*, *and Olea europaea* [21]. These bioactive compounds offer promising directions for developing novel anti-Echinococcus drugs. Our prior study demonstrated that H-2-168, a derivative of HM, exhibits potent anti-CE activity [8]. To further validate this finding, we employed a KM mouse model of hepatic CE infection. Results showed that H-2-168 significantly attenuated the progression of hepatic CE, evidenced by a marked reduction in liver cystic lesions, consistent with our initial hypothesis. Due to their favorable biocompatibility and biodegradability, PLGA nanoparticles are widely utilized for drug encapsulation and targeted delivery [22]. For instance, a 30-day oral administration of H1402 NPs at 100 mg/kg/day in a mouse model of hepatic alveolar echinococcosis (AE) reduced parasite mass (combined liver and larval weight) by 8.8% and decreased the average larval size by 89.9%, outperforming ABZ and free H1402 (*P* < 0.05) [23]. A preclinical study using a murine model of cystic echinococcosis demonstrated that FLBZ-loaded nanoparticles exhibited significantly enhanced efficacy in suppressing hydatid cyst development compared to free FLBZ [24]. In the current study, H-2-168-loaded NPs were applied for the first time in an animal treatment model. Our findings demonstrated enhanced parasiticidal efficacy in mice, aligning with previous reports indicating that nanoparticles can improve therapeutic outcomes through sustained drug delivery to lesions, enhanced bioavailability, and prolonged drug retention within target organs and cysts [24].

*E. granulosus* tapeworm infection elicits the liver’s secretion of abundant pro-inflammatory cytokines, initiating an inflammatory response [16]. Sustained inflammation, in turn, predisposes to peripheral tissue damage [25]. PLGA NPs are widely utilized for encapsulating anti-inflammatory drugs, and their anti-inflammatory properties have been validated across diverse models [26]. For instance, in a mouse model of malaria infection, oral administration of PLGA-curcumin more effectively prevented blood-brain barrier disruption and suppressed brain inflammatory cytokine mRNA expression compared to natural curcumin [27]. In this study, we observed that H8-PLGA-NPs significantly suppressed the mRNA levels of IL-6 and TNF-α, key indicators of hepatic inflammation, more potently than free H-2-168. These results are consistent with previous findings and further underscore the potential of H8-PLGA-NPs in treating hepatic CE by modulating inflammatory factors.

Echinococcus tapeworm infection triggers a hepatic inflammatory response and immune cell activation, both of which can induce liver fibrosis. On the one hand, the infection activates hepatic stellate cells (HSCs), prompting them to secrete abundant collagen, resulting in excessive extracellular matrix (ECM) deposition [28]. On the other hand, mechanical compression from growing encapsulated cysts causes local liver ischemia and tissue damage, further exacerbating the fibrotic process [29]. The TGF-β/smad3 pathway plays a pivotal role in liver fibrosis, and inhibiting its expression represents an effective strategy for alleviating this condition [30]. This study demonstrated that both H-2-168 and H8-PLGA-NPs could mitigate CE infection-induced liver fibrosis and injury, consistent with previous findings on HM. Notably, H8-PLGA-NPs exhibited a more pronounced effect, potentially attributable to their sustained-release characteristics. However, the specific protective mechanisms underlying this efficacy warrant further in-depth investigation.

We analyzed the shared metabolic pathways among the PSCs, the model group, the H-2-168 group, and the H8-PLGA-NPs group. The results revealed significant enrichment of 11 metabolic pathways, including aminoacyl-tRNA biosynthesis, tyrosine metabolism, tryptophan metabolism, and purine metabolism.Aminoacyl-tRNA synthetase (aaRS), a crucial enzyme for protein synthesis, is essential for parasite survival and reproduction. Previous studies have demonstrated that inhibiting aaRS activity disrupts parasite protein synthesis, thereby suppressing growth or inducing parasite death [31], findings highly congruent with our study’s results. Purine metabolites participate in fundamental biological processes such as energy production and DNA/RNA synthesis. Additionally, they function as extracellular signaling molecules, regulating matrix deposition and contributing to the fibrotic process. Evidence shows that blocking purine metabolite receptors reduces collagen formation and inhibits hepatic stellate cell activation in mouse models of liver fibrosis [32]. In mice with hepatic cystic echinococcosis, both tryptophan and tyrosine metabolism were significantly perturbed, collectively disrupting hepatic metabolic homeostasis [33]. These observations align with our current findings and suggest that these metabolic alterations are closely associated with liver function impairment and the inflammatory response triggered by parasitic infection. Therefore, the therapeutic effect of H8-PLGA-NPs against *E. granulosus* is likely mediated by the regulation of these metabolic pathways. Furthermore, the impact of liver worm infection on metabolic pathways, including carbon metabolism, pantothenic acid and coenzyme A biosynthesis, pyrimidine metabolism, and unsaturated fatty acid biosynthesis, provides valuable directions for identifying potential drug targets and elucidating therapeutic mechanisms.

Based on the particular structure of the cyst wall, we propose that the significantly enhanced therapeutic efficacy of H8-PLGA-NPs observed in this study may not result from the direct penetration of intact nanoparticles through the dense laminated layer of the cyst. The underlying mechanism is more likely attributed to the extensive accumulation of the nanocarrier in the pericystic region via the enhanced permeability and retention (EPR) effect, which establishes a sustained drug reservoir [34], coupled with its more potent destructive effect on germinal layer cells, potentially compromising the integrity of the cyst wall and creating favorable conditions for subsequent drug action [35]. Consequently, this delivery system enhances the intrinsic pharmacological activity of H-2-168 by optimizing the pharmacokinetic profile of the drug in the peri‑lesional area.

While the results of this study demonstrate promising anti-parasitic and anti-inflammatory activities, several limitations of this exploratory work must be acknowledged. First, the absence of a blank PLGA nanoparticle control group limits our ability to precisely quantify the respective contributions of the active drug H-2-168 and the nanocarrier itself to the overall therapeutic efficacy. Second, the sample size used in this study (n = 5 per group) should be understood within the context of the model‘s technical characteristics. Although the hepatic portal vein injection method is considered the gold standard for establishing secondary echinococcosis, it inherently carries a significant rate of acute procedural mortality. While this sample size strategy ensured that sufficient animals completed the treatment protocol, it may still limit the statistical power to detect more subtle biological effects, such as changes in specific immune cell subpopulations. Third, the 30-day treatment period provides preliminary sub-chronic toxicity data; however, comprehensive long-term toxicity, reproductive toxicity, and immunogenicity profiles of H8-PLGA-NPs remain to be established in future studies. Furthermore, this study has not yet addressed several key mechanistic and property-related questions, including: the in vivo biodistribution, retention time, and interactions with the host immune system of the nanoparticles; their pharmacokinetic and pharmacodynamic behaviors across species; the process stability and reproducibility of the nano-formulation during scale-up production; and the drug release kinetics under physiologically relevant pH conditions beyond the physiological pH 7.4 examined here (e.g., gastric pH 1.2 or inflammatory/tumor microenvironment pH ~ 6.8). An in-depth investigation of these issues will be an important direction for future research.

## 5. Conclusion

Overall, this study elucidates that H-2-168 is a novel anti-*E. granulosus* compound that exerts its efficacy by disrupting parasite energy homeostasis. H-2-168-loaded PLGA nanoparticles (H8-PLGA-NPs) significantly enhance drug enrichment at the lesion sites, improve the protoscolicidal effect against E. granulosus protoscoleces. More importantly, this nano-delivery system also exhibits anti-inflammatory activity, contributing to the modulation of the immune microenvironment at the infection site. Consequently, H8-PLGA-NPs intervene in disease progression on multiple levels by integrating potent direct anti-parasitic action with improved pharmacokinetic properties. These findings provide critical preclinical evidence for the development of nanotechnology-based combination therapeutic strategies against echinococcosis.

## Supporting information

S1 Table Quantitative PCR primers. (DOCX)

S2 Table Differentially expressed metabolites identified between the H-2-168 treatment group and the control group (in vitro). (DOCX)

S3 Table Differentially expressed metabolites identified between the Model and H-2-168 groups. (DOCX)

S4 Table The in vitro release fitting equation and the correlation coefficient of H8-PLGA-NPs. (DOCX)

S5 Table Differentially expressed metabolites identified between the H-2-168 and H8-PLGA-NPs groups. (DOCX)

S6 Table Determination of the recovery rate of H-2-168 from H8-PLGA-NPs. Data are mean ± SD (n = 3 independent experiments). (DOCX)

S1 Fig Calibration curve for the quantification of H-2-168. Data are mean ± SD (n = 3 independent experiments). Statistical significance: *P < 0.05, **P < 0.01 and ***P < 0.001.
(TIF)

S2 Fig (S2a) Chromatogram of H-2-168, (S2b), Chromatogram of H8-PLGA-NPs. (TIF)

S1 Text Test of the linear relationship of H-2-168. (DOCX)

S3 Fig Graphical Abstract: PLGA-Encapsulated Harmine Derivative H-2-168 could enhance the anti-echinococcosis efficacy and alleviate the inflammatory response and liver fibrosis in Echinococcus‑infected mice. (TIF)
