# Supplementary material for: PLGA - encapsulated harmine derivative H-2-168: A promising therapeutic agent for mitigating liver damage in hepatic hydatid disease
Source: PLoS Negl Trop Dis. 2026 Jul 24;20(7):e0014483. doi: 10.1371/journal.pntd.0014483 (PMC13399313; doi:10.1371/journal.pntd.0014483)
Supplement: S1 Table — (DOCX) [file pntd.0014483.s001.docx]

**S1 Table**. Quantitative PCR Primers.

| **Primers Name** | | **PrimersSequences**  **(5′ to3′)** | **Length(bp)** | |
| --- | --- | --- | --- | --- |
| Mus-β-actin | Forward | CACCACACCTTCTACAAC | | 18 |
|  | Reverse | TCTGGGTCATCTTCTCAC | | 18 |
| Mus-MyD88 | Forward | GCAGAACCAGGAGTCCGAGAAG | | 22 |
|  | Reverse | GATGCCTCCCAGTTCCTTTGTTTG | | 24 |
| Mus-NF-κB p65 | Forward | ATGGGAAACCGTATGAGCCTGTG | | 23 |
|  | Reverse | AGTTGTAGCCTCGTGTCTTCTGTC | | 24 |
| Mus-IL-9 | Forward | GATGCGGCTGATTGTTT | | 17 |
|  | Reverse | CTCGTGCTCACTGTGGAGT | | 19 |
| Mus-α-SMA | Forward | TTCGTGACTACTGCCGAGC | | 19 |
|  | Reverse | CCCTGGTCCCTCTGGAAATG | | 20 |
| Mus-Collagen I | Forward | GGACCTTTGCCCCCTTCTTT | | 20 |
|  | Reverse | GGTTGTCTCCTGCGACTTCA | | 20 |
| Mus-Actin | Forward | CACCACACCTTCTACAAC | | 18 |
|  | Reverse | TCTGGGTCATCTTCTCAC | | 18 |
