# Supplementary material for: PLGA - encapsulated harmine derivative H-2-168: A promising therapeutic agent for mitigating liver damage in hepatic hydatid disease
Source: PLoS Negl Trop Dis. 2026 Jul 24;20(7):e0014483. doi: 10.1371/journal.pntd.0014483 (PMC13399313; doi:10.1371/journal.pntd.0014483)
Supplement: S2 Table — (DOCX) [file pntd.0014483.s002.docx]

**S2 Table.** Differentially expressed metabolites identified between the H-2-168 treatment group and the control group (in vitro).

| **Number** | **Name** | **m/z** | **VIP** | ***P*** | **Ion Mode** |
| --- | --- | --- | --- | --- | --- |
| 1 | m-Cresol | 109.1012 | 2.041121236 | 0.007940631 | pos |
| 2 | Hydroquinone | 110.0193 | 1.709795412 | 0.033102913 | pos |
| 3 | Cytosine | 112.0504 | 1.70895087 | 0.035623637 | pos |
| 4 | Dihydrouracil | 115.039 | 1.839895096 | 0.02291763 | pos |
| 5 | Picolinic acid | 124.0865 | 1.837737378 | 0.019481104 | pos |
| 6 | D-Ornithine | 133.0972 | 1.746578836 | 0.046113707 | pos |
| 7 | 3-Hydroxymethylglutaric acid | 144.9821 | 1.886104503 | 0.015657301 | pos |
| 8 | Se-Methylselenocysteine | 182.9852 | 2.194849542 | 0.005789383 | pos |
| 9 | Sorbitol | 182.9849 | 1.82589806 | 0.017975682 | pos |
| 10 | gamma-Glutamylalanine | 219.0975 | 1.7041838 | 0.039356033 | pos |
| 11 | N-Acetyl-D-galactosamine | 222.0972 | 2.185831096 | 0.00074075 | pos |
| 12 | 1-Hexadecanol | 243.1823 | 1.771968478 | 0.026029233 | pos |
| 13 | 3,3'-Dimethoxybenzidine | 245.1289 | 2.18908944 | 0.001028153 | pos |
| 14 | Sotalol | 255.1136 | 1.795055949 | 0.024069363 | pos |
| 15 | Nicotinamide riboside | 255.0982 | 1.712501714 | 0.035683051 | pos |
| 16 | (2R,3R)-3-Methylglutamyl-5-semialdehyde-N6-lysine | 274.1866 | 1.818865967 | 0.023718731 | pos |
| 17 | 5'-Methylthioadenosine | 298.096 | 1.731241131 | 0.03753459 | pos |
| 18 | N-Acetylneuraminic acid | 310.1124 | 1.984371813 | 0.007570292 | pos |
| 19 | Oleoylethanolamide | 326.305 | 1.633424386 | 0.049022772 | pos |
| 20 | Sucrose | 343.2952 | 2.24048349 | 0.000674369 | pos |
| 21 | Anandamide | 348.2899 | 1.905217376 | 0.018605677 | pos |
| 22 | Alpha-Linolenoyl ethanolamide | 349.2931 | 1.901553712 | 0.017695139 | pos |
| 23 | Riboflavin | 377.1443 | 1.973704584 | 0.008388622 | pos |
| 24 | S-Hexyl-glutathione | 391.2829 | 1.752099118 | 0.044706705 | pos |
| 25 | Allocholic acid | 408.3656 | 1.785818501 | 0.023156329 | pos |
| 26 | Methylprednisolone acetate | 416.2322 | 1.85387969 | 0.013415659 | pos |
| 27 | 2-Ketobutyric acid | 101.024 | 2.415306818 | 2.33383E-05 | neg |
| 28 | Oxalacetic acid | 112.9856 | 1.894190472 | 0.023915169 | neg |
| 29 | 1H-Indole-3-carboxaldehyde | 144.0457 | 1.768995682 | 0.041424746 | neg |
| 30 | L-Methionine | 148.0437 | 1.87152828 | 0.033828865 | neg |
| 31 | D-Mannose | 179.0563 | 2.153347473 | 0.003747899 | neg |
| 32 | Indolelactic acid | 204.0666 | 2.070358314 | 0.007059539 | neg |
| 33 | Deoxyinosine | 251.0794 | 1.895291819 | 0.024978562 | neg |
| 34 | Deoxyguanosine | 266.0894 | 1.881884845 | 0.032901153 | neg |
| 35 | 2-Methoxyestrone | 300.1809 | 1.974388159 | 0.016360094 | neg |
| 36 | Arachidonic acid | 303.2328 | 2.089690408 | 0.008065477 | neg |
| 37 | 11Z-Eicosenoic acid | 310.2836 | 1.806748502 | 0.042055754 | neg |
| 38 | CMP | 322.0436 | 1.773625076 | 0.039572901 | neg |
| 39 | Melibiitol | 344.136 | 2.207878139 | 0.002372203 | neg |
