# Supplementary material for: PLGA - encapsulated harmine derivative H-2-168: A promising therapeutic agent for mitigating liver damage in hepatic hydatid disease
Source: PLoS Negl Trop Dis. 2026 Jul 24;20(7):e0014483. doi: 10.1371/journal.pntd.0014483 (PMC13399313; doi:10.1371/journal.pntd.0014483)
Supplement: S3 Table — (DOCX) [file pntd.0014483.s003.docx]

**S3 Table.** Differentially expressed metabolites identified between the Model and H-2-168 groups

| **Number** | **Name** | **m/z** | **VIP** | ***P*** | **Ion Mode** |
| --- | --- | --- | --- | --- | --- |
| 1 | m-Cresol | 109.1013 | 1.5664666 | 0.019541 | pos |
| 2 | 2-Aminophenol | 110.0192 | 1.920160286 | 0.000534 | pos |
| 3 | Catechol | 111.0208 | 1.984535555 | 6.64E-05 | pos |
| 4 | 2-Heptanone | 113.9626 | 1.872005149 | 0.000858 | pos |
| 5 | Niacinamide | 123.0542 | 1.522899279 | 0.021564 | pos |
| 6 | Phloroglucinol | 127.0378 | 1.384497919 | 0.040202 | pos |
| 7 | Maltol | 127.0379 | 1.412355247 | 0.028609 | pos |
| 8 | 1,2,3-Trihydroxybenzene | 127.0381 | 1.471397272 | 0.016529 | pos |
| 9 | Ketoleucine | 130.9663 | 2.051678702 | 4.35E-06 | pos |
| 10 | Leucine | 132.1006 | 1.652790677 | 0.00696 | pos |
| 11 | Indolin-2-one | 134.0632 | 1.50258428 | 0.016038 | pos |
| 12 | Acetylphosphate | 139.9824 | 2.014214882 | 3.52E-05 | pos |
| 13 | L-Glutamine | 146.0826 | 1.695591504 | 0.007685 | pos |
| 14 | L-Methionine | 150.0586 | 1.957783104 | 0.000138 | pos |
| 15 | 3-Hydroxyanthranilate | 153.0408 | 1.455787506 | 0.045386 | pos |
| 16 | 2-Biphenylol | 153.0661 | 1.596642537 | 0.006682 | pos |
| 17 | Imidazol-5-yl-pyruvate | 154.042 | 1.790090717 | 0.002893 | pos |
| 18 | L-Histidine | 156.0768 | 1.349228182 | 0.037934 | pos |
| 19 | Uracil 5-carboxylate | 156.9658 | 1.84504243 | 0.004339 | pos |
| 20 | Pterin | 164.057 | 1.698320526 | 0.004602 | pos |
| 21 | Eugenol | 165.0912 | 1.525462747 | 0.012143 | pos |
| 22 | 4-Hydroxyphenylglyoxylate | 166.0367 | 1.852862966 | 0.003795 | pos |
| 23 | (R)-2-O-Sulfolactate | 169.9786 | 2.066025741 | 1.2E-06 | pos |
| 24 | 4-Quinolinecarboxylic acid | 172.9575 | 1.470805154 | 0.024294 | pos |
| 25 | 3,4-Dihydroxymandelic acid | 184.984 | 1.945407679 | 0.000149 | pos |
| 26 | Undecanoic acid | 186.9578 | 1.991303177 | 5.05E-05 | pos |
| 27 | (3S)-6-Acetamido-3-aminohexanoate | 188.1286 | 1.491579471 | 0.032095 | pos |
| 28 | N8-Acetylspermidine | 188.1761 | 1.631969358 | 0.006183 | pos |
| 29 | 5,6-Dihydroxy-3-methyl-2-oxo-1,2,5,6-tetrahydroquinoline | 193.068 | 1.759716608 | 0.002245 | pos |
| 30 | Neocnidilide | 195.1384 | 1.641818617 | 0.01784 | pos |
| 31 | Dodecanoic acid | 200.9709 | 1.548924182 | 0.022304 | pos |
| 32 | Thiabendazole | 202.0446 | 1.892423163 | 0.000575 | pos |
| 33 | (+)-7-Isojasmonic acid | 211.133 | 2.055727058 | 7.56E-07 | pos |
| 34 | Dethiobiotin | 215.1394 | 1.76915355 | 0.003051 | pos |
| 35 | Propionylcarnitine | 218.1389 | 1.897018185 | 0.000465 | pos |
| 36 | Butyryl-L-carnitine | 232.1548 | 1.459385924 | 0.026028 | pos |
| 37 | 1-Hexadecanol | 243.1824 | 1.39009666 | 0.042037 | pos |
| 38 | Lumichrome | 243.088 | 1.641272019 | 0.011315 | pos |
| 39 | Uridine | 245.0771 | 1.630590654 | 0.02307 | pos |
| 40 | Daidzein | 255.0652 | 1.871552144 | 0.000438 | pos |
| 41 | Adenosine | 268.104 | 1.337200813 | 0.046344 | pos |
| 42 | 17a-Estradiol | 272.1863 | 1.708996738 | 0.014276 | pos |
| 43 | 4-Hydroxycinnamoylagmatine | 276.1442 | 1.459497042 | 0.026847 | pos |
| 44 | (5-L-Glutamyl)-L-glutamate | 277.1028 | 1.637643417 | 0.008243 | pos |
| 45 | Oleamide | 282.2781 | 1.408066599 | 0.036067 | pos |
| 46 | Ophthalmate | 290.1345 | 1.688797025 | 0.00384 | pos |
| 47 | Trimethoprim | 291.1449 | 1.428304482 | 0.032379 | pos |
| 48 | N-Acetyl-a-neuraminic acid | 292.1016 | 1.72949106 | 0.002376 | pos |
| 49 | Sphingosine | 300.2895 | 1.433113463 | 0.036931 | pos |
| 50 | 8-HETE | 303.231 | 1.360388827 | 0.042427 | pos |
| 51 | Glutathione | 307.083 | 1.452133655 | 0.033115 | pos |
| 52 | 2,3-Dinor-8-iso prostaglandin F2alpha | 309.2064 | 1.864903972 | 0.000536 | pos |
| 53 | 13(S)-HpOTrE | 311.2211 | 1.547467514 | 0.029551 | pos |
| 54 | Rosmarinic acid | 360.2383 | 1.899495982 | 0.000372 | pos |
| 55 | Nitrendipine | 361.1386 | 1.484679712 | 0.029745 | pos |
| 56 | Docosatetraenoyl Ethanolamide | 376.3182 | 1.634901621 | 0.008797 | pos |
| 57 | S-Glutathionyl-L-cysteine | 426.098 | 1.620147415 | 0.009277 | pos |
| 58 | Folic acid | 442.1457 | 1.537157357 | 0.01862 | pos |
| 59 | Antibiotic G-418 | 497.2743 | 1.44769681 | 0.022447 | pos |
| 60 | L-Olivosyl-oleandolide | 516.3079 | 1.460584417 | 0.037599 | pos |
| 61 | Avermectin A1b aglycone | 567.3318 | 1.423218688 | 0.036829 | pos |
| 62 | Protoporphyrinogen IX | 568.3468 | 1.292934093 | 0.04946 | pos |
| 63 | Avermectin B1b aglycone | 570.3607 | 1.570926693 | 0.018808 | pos |
| 64 | 5-Oxoavermectin ''2b'' aglycone | 586.331 | 1.423197542 | 0.032504 | pos |
| 65 | 5-Oxoavermectin ''2a'' aglycone | 600.322 | 1.834310148 | 0.001048 | pos |
| 66 | Succinic acid semialdehyde | 101.0238 | 1.726241612 | 0.009103 | neg |
| 67 | (R)-3-Hydroxybutyric acid | 103.04 | 1.507642023 | 0.036337 | neg |
| 68 | 1-Naphthylamine | 123.902 | 1.832052743 | 0.003972 | neg |
| 69 | Protocatechuic acid | 153.0205 | 1.966840652 | 0.00184 | neg |
| 70 | Allantoin | 157.0365 | 1.5735125 | 0.03173 | neg |
| 71 | Azelaic acid | 169.0869 | 1.493648281 | 0.036354 | neg |
| 72 | DL-Glycerol 1-phosphate | 171.0063 | 1.703532071 | 0.013181 | neg |
| 73 | Shikimic acid | 173.0452 | 1.612986222 | 0.023687 | neg |
| 74 | Hippuric acid | 178.0504 | 1.818522999 | 0.005372 | neg |
| 75 | 4-Pyridoxic acid | 182.0459 | 1.809657778 | 0.00731 | neg |
| 76 | Ecgonine | 184.0979 | 1.66227643 | 0.017797 | neg |
| 77 | 3,7-Dimethyluric acid | 195.0512 | 1.669241118 | 0.012803 | neg |
| 78 | alpha-D-Ribose 1-phosphate | 229.0117 | 1.641831821 | 0.01601 | neg |
| 79 | Inosine | 267.0735 | 1.517089832 | 0.030933 | neg |
| 80 | Epiandrosterone | 271.2287 | 1.556033634 | 0.04721 | neg |
| 81 | 6-Phospho-2-dehydro-D-gluconate | 273.0014 | 1.438615641 | 0.041456 | neg |
| 82 | 9,10-Epoxyoctadecenoic acid | 295.2293 | 1.873202134 | 0.003698 | neg |
| 83 | Sclareol | 308.2669 | 1.729203749 | 0.007758 | neg |
| 84 | 9,10-12,13-Diepoxyoctadecanoate | 311.2243 | 1.874602267 | 0.003641 | neg |
| 85 | 9(S)-HPODE | 311.2243 | 1.639739335 | 0.024115 | neg |
| 86 | Levonorgestrel | 311.2011 | 1.667978037 | 0.019661 | neg |
| 87 | 12-Keto-tetrahydro-leukotriene B4 | 317.211 | 1.912936647 | 0.009534 | neg |
| 88 | 11,12-EET | 319.2263 | 1.603762582 | 0.015335 | neg |
| 89 | dTMP | 321.0437 | 2.035160291 | 0.0006 | neg |
| 90 | 5,6-DHET | 337.2403 | 2.056585063 | 0.000195 | neg |
| 91 | Prostaglandin F2a | 353.2331 | 1.477595336 | 0.048994 | neg |
