# Supplementary material for: PLGA - encapsulated harmine derivative H-2-168: A promising therapeutic agent for mitigating liver damage in hepatic hydatid disease
Source: PLoS Negl Trop Dis. 2026 Jul 24;20(7):e0014483. doi: 10.1371/journal.pntd.0014483 (PMC13399313; doi:10.1371/journal.pntd.0014483)
Supplement: S4 Table — (DOCX) [file pntd.0014483.s004.docx]

**S4 Table.** Fitting equations and correlation coefficients for the *in vitro* release of H8-PLGA-NPs

| **Drug release model** | **Equation** | **R^2^** |
| --- | --- | --- |
| Zero-order equation | *Q*=34.9454+0.8332 *t* | 0.7209 |
| First-order equation | *Q*=91.57*(1-e^-0.11^ *t*) | 0.9600 |
| Higuchi | *Q*=9.6116 *t*^1/2^+17.2381 | 0.8958 |
| Riter-peppas | *Q*=24.7672 *t*^0.3201^ | 0.9497 |
