# Supplementary material for: PLGA - encapsulated harmine derivative H-2-168: A promising therapeutic agent for mitigating liver damage in hepatic hydatid disease
Source: PLoS Negl Trop Dis. 2026 Jul 24;20(7):e0014483. doi: 10.1371/journal.pntd.0014483 (PMC13399313; doi:10.1371/journal.pntd.0014483)
Supplement: S5 Table — (DOCX) [file pntd.0014483.s005.docx]

**S5 Table.** Differentially expressed metabolites identified between the H-2-168 and H8-PLGA-NPs groups

| **Number** | **Name** | **m/z** | **VIP** | ***P*** | **Ion Mode** |
| --- | --- | --- | --- | --- | --- |
| 1 | (S)-2-Methylbutanal | 104.1074 | 1.808082 | 4.46E-06 | pos |
| 2 | 2-Aminoisobutyric acid | 104.071 | 1.981396 | 2.42E-10 | pos |
| 3 | Hydroxylaminobenzene | 110.0601 | 1.857717 | 8.14E-07 | pos |
| 4 | 4,5-Dimethylthiazole | 114.0663 | 1.08753 | 0.033656 | pos |
| 5 | 2-Furancarboxaldehyde | 114.055 | 1.567307 | 0.000481 | pos |
| 6 | N(1)-isopropyl-2-methylpropan-1,2-diamine | 114.0914 | 1.022454 | 0.048138 | pos |
| 7 | Isovaline | 118.0858 | 1.038494 | 0.042911 | pos |
| 8 | AI3-34796 | 118.1223 | 1.89581 | 1.61E-07 | pos |
| 9 | Glutaral | 118.0858 | 1.301908 | 0.014377 | pos |
| 10 | Carnitine | 118.1203 | 1.726659 | 3.6E-05 | pos |
| 11 | L-Aspartate-semialdehyde | 118.0509 | 1.989362 | 1.42E-07 | pos |
| 12 | 4-Oxo-1-(3-pyridyl)-1-butanone | 120.0804 | 1.206883 | 0.022733 | pos |
| 13 | Nona-2,4,6-trienal | 120.0805 | 1.037991 | 0.042973 | pos |
| 14 | Styrene | 122.0963 | 1.345227 | 0.005438 | pos |
| 15 | Phenylhydrazine | 126.1027 | 1.385662 | 0.003726 | pos |
| 16 | Oxoglutaric acid | 129.0184 | 1.741769 | 2.45E-05 | pos |
| 17 | trans-1,2-Dihydrobenzene-1,2-diol | 130.0858 | 1.012722 | 0.049423 | pos |
| 18 | 1,4-Diazabicyclo[2.2.2]octane | 130.0492 | 1.390024 | 0.003627 | pos |
| 19 | (Z)-4-Hepten-1-ol | 132.1005 | 2.001575 | 9.59E-12 | pos |
| 20 | 3-Hydroxycyclohexanone | 132.1005 | 1.135496 | 0.025302 | pos |
| 21 | Phenylacetaldoxime | 136.0761 | 1.570594 | 0.000459 | pos |
| 22 | 1-Chloro-2-nitrobenzene | 139.9881 | 1.875252 | 4.25E-07 | pos |
| 23 | 2,6-Dimethyl-2,4-heptadiene | 142.1224 | 1.327612 | 0.006092 | pos |
| 24 | Naphthalene-1,2-diol | 143.0484 | 1.078701 | 0.035498 | pos |
| 25 | 1,2,3,5-Benzenetetrol | 143.0336 | 1.66605 | 0.000107 | pos |
| 26 | 2,5-Diethyltetrahydrofuran | 146.0817 | 1.154929 | 0.021574 | pos |
| 27 | Pantolactone | 146.1175 | 1.881538 | 3.14E-07 | pos |
| 28 | (S)-2-Aceto-2-hydroxybutanoic acid | 147.0655 | 1.638283 | 0.000174 | pos |
| 29 | L-Lysine | 147.112 | 1.903218 | 1.14E-07 | pos |
| 30 | Phenylpyruvate | 147.0441 | 1.500061 | 0.001137 | pos |
| 31 | dihydro-3-hydroxy-4,4-dimethyl- 2(3H)-Furanone | 148.0967 | 1.089966 | 0.044555 | pos |
| 32 | Methionine sulfoxide | 148.0427 | 1.443049 | 0.002174 | pos |
| 33 | 2,6-Diethylaniline | 150.1274 | 1.655314 | 0.000127 | pos |
| 34 | Ethionamide | 150.059 | 1.789689 | 7.24E-06 | pos |
| 35 | (R)-Campholenic aldehyde | 153.091 | 1.992433 | 6.19E-11 | pos |
| 36 | 4-Trimethylammoniobutanal | 153.1137 | 2.028924 | 1.29E-16 | pos |
| 37 | Xanthine | 153.0407 | 1.698017 | 0.000758 | pos |
| 38 | trans-Ocimene | 154.159 | 2.002598 | 6.94E-12 | pos |
| 39 | Glycylglycine | 155.0821 | 1.564742 | 0.000484 | pos |
| 40 | O-Phosphohomoserine | 156.0421 | 1.900505 | 1.24E-07 | pos |
| 41 | Ethyl trans-p-methoxycinnamate | 163.1118 | 1.742026 | 2.59E-05 | pos |
| 42 | cis-6-Nitro-p-mentha-1(7),2-diene | 164.0676 | 1.840473 | 1.6E-06 | pos |
| 43 | octanoate | 166.0972 | 1.942579 | 1.01E-08 | pos |
| 44 | 2-Amino-3-cyclohexylpropanoic acid | 171.9928 | 1.408244 | 0.00772 | pos |
| 45 | 5-Methyl-2-(1-methylethyl)-cyclohexanone | 172.1697 | 1.462736 | 0.001725 | pos |
| 46 | N-Acetylleucine | 174.1137 | 1.83473 | 1.91E-06 | pos |
| 47 | Isobutanal oxime | 175.1443 | 1.696374 | 0.000599 | pos |
| 48 | Citrulline | 176.1031 | 1.998441 | 2.08E-11 | pos |
| 49 | Hept-trans-2-en-1-yl acetate | 179.1066 | 1.268862 | 0.009838 | pos |
| 50 | Caffeyl alcohol | 184.0973 | 1.251075 | 0.011364 | pos |
| 51 | D-Erythrose 4-phosphate | 183.9878 | 1.562245 | 0.002129 | pos |
| 52 | L-Glutamine | 185.1653 | 1.185492 | 0.017868 | pos |
| 53 | Benzyl isothiocyanate | 188.1285 | 1.488185 | 0.001265 | pos |
| 54 | Methyl indole-3-acetate | 190.0861 | 1.682026 | 0.000443 | pos |
| 55 | Pyridoxal | 190.05 | 1.991625 | 6.64E-11 | pos |
| 56 | Pyridoxine | 192.0621 | 1.314862 | 0.006637 | pos |
| 57 | 3,5-DICHLOROCATECHOL | 195.9932 | 1.06992 | 0.04812 | pos |
| 58 | Coniferaldehyde | 201.0506 | 1.633812 | 0.000193 | pos |
| 59 | Phosphoroselenoic acid | 200.9972 | 1.126567 | 0.026049 | pos |
| 60 | 1-Acetyl-4-phenyl-1,2,4-triazolidine-3,5-dione | 202.1199 | 1.162362 | 0.021158 | pos |
| 61 | Heteropyrithiamine | 202.1786 | 1.076063 | 0.03512 | pos |
| 62 | OR-1896 | 202.029 | 1.867773 | 6.46E-07 | pos |
| 63 | Asymmetric dimethylarginine | 203.1503 | 1.129713 | 0.025734 | pos |
| 64 | Acetyl-L-carnitine | 204.123 | 1.604639 | 0.001335 | pos |
| 65 | L-Tryptophan | 205.0971 | 1.167366 | 0.020087 | pos |
| 66 | Acetylphenylalanine | 208.0971 | 1.050536 | 0.040938 | pos |
| 67 | L-Kynurenine | 209.0921 | 1.383716 | 0.00696 | pos |
| 68 | Lotaustralin | 218.1388 | 1.455896 | 0.001801 | pos |
| 69 | 8-Amino-7-oxononanoic acid | 226.1802 | 2.002054 | 8.59E-12 | pos |
| 70 | 1-Ethylhexyl tiglate | 230.2475 | 1.093587 | 0.032004 | pos |
| 71 | Isoavocadienofuran | 230.1831 | 1.055151 | 0.039433 | pos |
| 72 | Butyrylcarnitine | 232.1543 | 1.760689 | 1.53E-05 | pos |
| 73 | Glycerylphosphorylethanolamine | 233.092 | 2.03205 | 1.18E-12 | pos |
| 74 | 4-Hydroxy tolbutamide | 243.1831 | 1.096954 | 0.030809 | pos |
| 75 | Gamma-Glutamylvaline | 247.1285 | 1.9678 | 1.28E-09 | pos |
| 76 | 16-Hydroxyhexadecanoic acid | 255.2321 | 1.74138 | 2.39E-05 | pos |
| 77 | 3-Dehydrosphinganine | 256.3 | 1.150742 | 0.033692 | pos |
| 78 | Glycerophosphocholine | 258.1101 | 1.613195 | 0.001538 | pos |
| 79 | Gamma-glutamyl-leucine | 261.1445 | 1.941666 | 1.22E-08 | pos |
| 80 | 3-Cyclohexyldodecane | 270.3155 | 1.094733 | 0.046855 | pos |
| 81 | Chymopapain | 271.04 | 1.020329 | 0.048598 | pos |
| 82 | 4-Methylphenyl dodecanoate | 273.2535 | 1.239474 | 0.012585 | pos |
| 83 | alpha-Santalyl acetate | 280.2632 | 1.985274 | 1.41E-10 | pos |
| 84 | Xanthosine | 285.0824 | 1.140102 | 0.024037 | pos |
| 85 | FA 18_1 | 300.2894 | 1.198791 | 0.025566 | pos |
| 86 | Thiamine | 304.2106 | 1.266333 | 0.010036 | pos |
| 87 | N-acetylneuraminate | 310.1131 | 1.248813 | 0.011118 | pos |
| 88 | 3-Hydroxy-1-phenyl-1-octadecanone | 317.2472 | 1.761438 | 1.49E-05 | pos |
| 89 | 5alpha-Pregnan-20alpha-ol-3-one | 319.2633 | 2.030676 | 2.13E-17 | pos |
| 90 | 9alpha-Hydroxyandrosta-1,4-diene-3,17-dione | 323.162 | 1.51789 | 0.00377 | pos |
| 91 | (S)-2-Methylbutanal | 104.1074 | 1.808082 | 4.46E-06 | pos |

"Note. The top 91 differentially expressed metabolites between the H-2-168 and H8-PLGA-NPs groups."
