# Supplementary material for: PLGA - encapsulated harmine derivative H-2-168: A promising therapeutic agent for mitigating liver damage in hepatic hydatid disease
Source: PLoS Negl Trop Dis. 2026 Jul 24;20(7):e0014483. doi: 10.1371/journal.pntd.0014483 (PMC13399313; doi:10.1371/journal.pntd.0014483)
Supplement: S6 Table — (DOCX) [file pntd.0014483.s006.docx]

**S6 Table.** Experimental Results of Recovery Rate

| Dosage amount（µg） | Measured quantity（µg） | Recall rate（%） | Average recovery rate（%） | RSD（%） |
| --- | --- | --- | --- | --- |
| 32.24 | 73.52 | 101.35 |  |  |
|  | 72.02 | 99.28 | 99.71 | 1.48 |
|  | 71.45 | 98.50 |  |  |
| 40.30 | 81.32 | 100.89 |  |  |
|  | 80.56 | 99.95 | 99.38 | 1.74 |
|  | 78.53 | 97.43 |  |  |
| 48.36 | 88.52 | 99.84 |  |  |
|  | 89.56 | 101.02 | 100.80 | 0.87 |
|  | 90.04 | 101.56 |  |  |
