# Supplementary figures and images for: PLGA - encapsulated harmine derivative H-2-168: A promising therapeutic agent for mitigating liver damage in hepatic hydatid disease
Source: PLoS Negl Trop Dis. 2026 Jul 24;20(7):e0014483. doi: 10.1371/journal.pntd.0014483 (PMC13399313; doi:10.1371/journal.pntd.0014483)

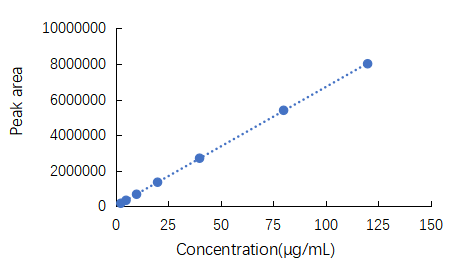

Supplement: S1 Fig — Data are mean ± SD (n = 3 independent experiments). Statistical significance: *P < 0.05, **P < 0.01 and ***P < 0.001. (TIF) [file pntd.0014483.s007.tif]

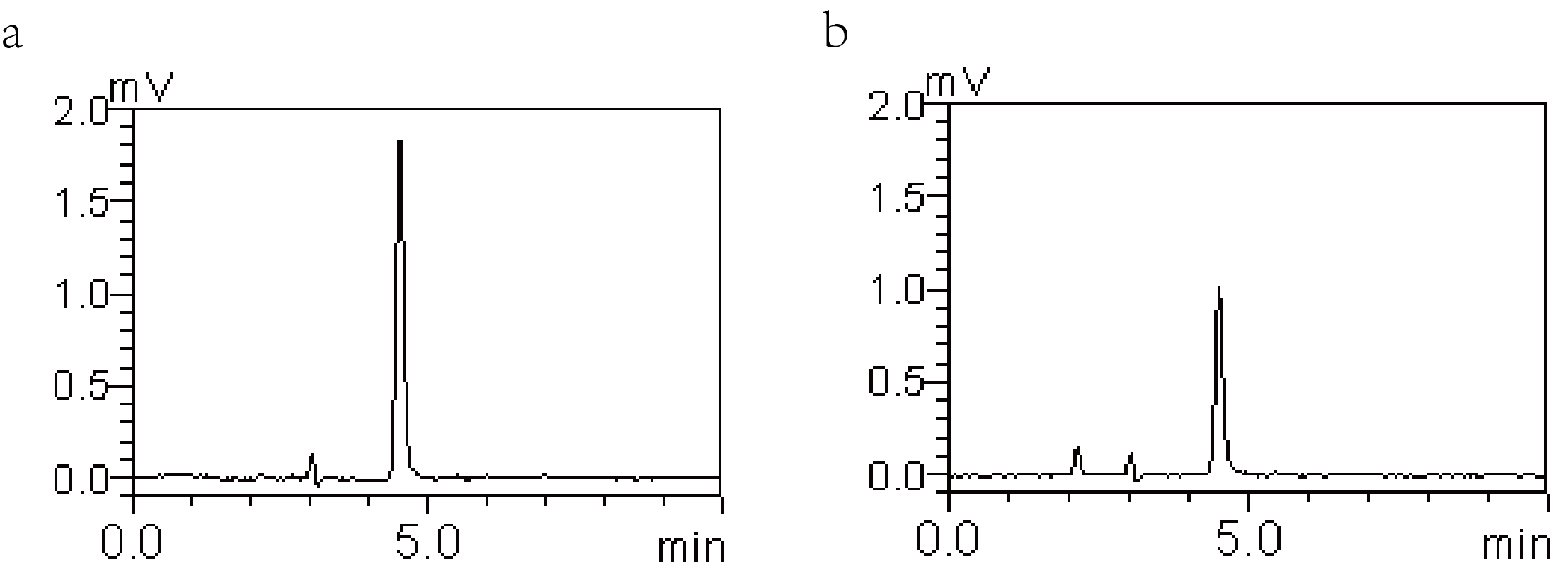

Supplement: S2 Fig — (TIF) [file pntd.0014483.s008.tif]

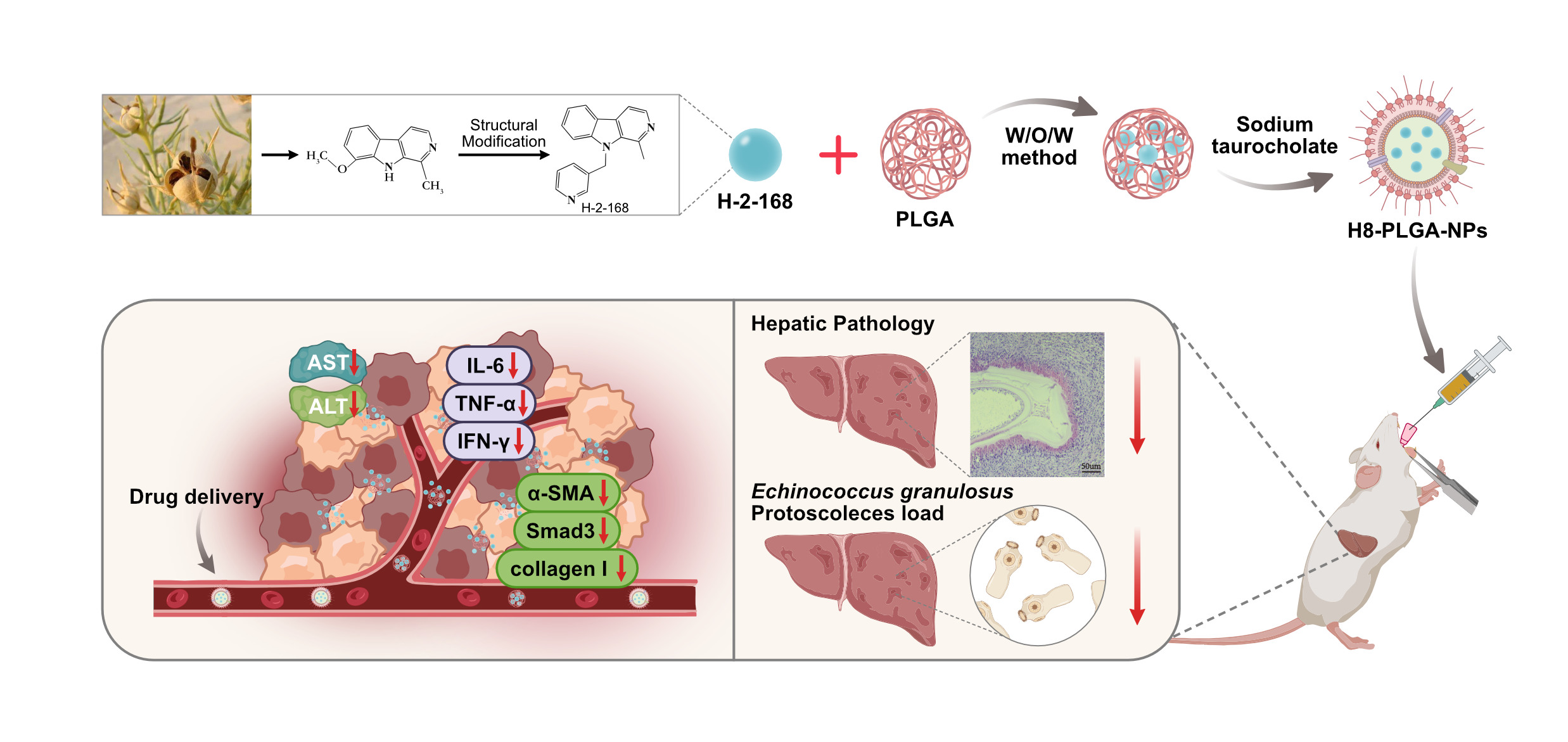

Supplement: S3 Fig — (TIF) [file pntd.0014483.s010.tif]
