# Supplementary material for: PLGA - encapsulated harmine derivative H-2-168: A promising therapeutic agent for mitigating liver damage in hepatic hydatid disease
Source: PLoS Negl Trop Dis. 2026 Jul 24;20(7):e0014483. doi: 10.1371/journal.pntd.0014483 (PMC13399313; doi:10.1371/journal.pntd.0014483)
Supplement: S1 Text — (DOCX) [file pntd.0014483.s009.docx]

**Test of the linear relationship of H-2-168**

Precisely weighed 5.00 mg of the H-2-168 reference standard, transferred it into a 25 mL volumetric flask, dissolved with methanol, and diluted to volume, thereby preparing a stock solution with a mass concentration of 200.0 µg/mL. Precisely measured 0.0625, 0.125, 0.25, 0.5, 1.0, 2.0, and 3.0 volumes of the stock solution into 5 mL volumetric flasks, and made up the volume with methanol to obtain a series of gradient concentration solutions. Using concentration as the abscissa and the peak area of derivative H8 as the ordinate, perform linear regression, obtaining the regression equation: Y = 66894X + 12345 (r = 0.9999) (**S1 Fig.** ), indicating that the linear relationship of H-2-168 is good within the range of 2.5 µg/mL - 120 µg/mL.
